# Upscaling cervical cancer screening and treatment for women living with HIV at a rural referral hospital in Tanzania: protocol of a before-and-after study exploring HPV testing and novel diagnostics

**DOI:** 10.1186/s12913-023-09113-3

**Published:** 2023-03-10

**Authors:** Ivana Di Salvo, Dorcas Mnzava, Giovanni Jacopo Nicoletti, Elizabeth Senkoro, Robert C. Ndege, Dorothy J. Huang, Nathalia Tobias Makunja, George I. Kassiga, Andreas M. Kaufmann, Maja Weisser, André B. Kind

**Affiliations:** 1 Colposcopy Unit, Department of Gynaecology and Gynaecologic Oncology, University Hospital of Basel, Spitalstrasse 21, Basel, 4031 Switzerland; 2 Chronic Disease Clinic, Department for Interventions and Clinical Trials, Ifakara Health Institute, Ifakara, United Republic of Tanzania; 3 Department of Medicine, Swiss Tropical and Public Health Institute, Basel, Switzerland; 4 University of Basel, Basel, Switzerland; 5 Saint Francis Referral Hospital, Ifakara, Tanzania; 6 Department of Obstetrics and Gynecology, St. Francis Referral District Hospital, Ifakara, Tanzania; 7 Charité – Universitätsmedizin Berlin, corporate member of Freie Universität Berlin and Humboldt-Universität zu Berlin, Department of Gynaecology, HPV Laboratory, Berlin, Germany; 8 Department of Infectious Diseases & Hospital Epidemiology, University Hospital of Basel, Basel, Switzerland

**Keywords:** Human papillomavirus, Mobile colposcopy, Cervical cancer, Cervical dysplasia, Women living with HIV, LEEP, Sub-Saharan Africa, Screening coverage, Thermal ablation, Tanzania

## Abstract

**Background:**

Cervical cancer (CC) is nearly always caused by persistent human papillomavirus (HPV) infection. It is the most common cancer among women living with HIV (WLWH) and is the leading cause of cancer-related death in women in East Africa, with 10,241 new cases reported in Tanzania in 2020. In 2019, the World Health Organization (WHO) presented a global strategy for the elimination of CC as a public health problem, proposing targets to meet by 2030 for HPV vaccine coverage (90% of all 15-year-old girls), CC screening (70% of all women once at 35 and again at 45 years of age) and treatment delivery, to be scaled at national and subnational levels with a context-sensitive approach. This study aims to evaluate the upscaling of screening and treatment services at a rural referral hospital in Tanzania in order to address the second and third WHO targets.

**Methods:**

This is an implementation study with a before-and-after design performed at St. Francis Referral Hospital (SFRH) in Ifakara (south-central Tanzania). CC screening and treatment services are integrated within the local HIV Care and Treatment Center (CTC). The standard of care, consisting of visualization of the cervix with acetic acid (VIA) and cryotherapy has been up-scaled with self-sampled HPV testing and also involved the introduction of mobile colposcopy, thermal ablation and loop electrosurgical excision procedure (LEEP). Participants are WLWH aged 18 to 65 years. Outcome measures included the percentage of women screened, HPV prevalence and genotype, and adherence to screening, treatment and follow-up plan. Additionally, we will explore the performance of novel diagnostic tests (QG-MPH®, Prevo-Check® and PT Monitor®), which share the features of being manageable and inexpensive, and thus a potential tool for effective triage in HPV high-prevalence cohorts.

**Discussion:**

The study will provide relevant information about HPV prevalence and persistence, as well as reproductive and lifestyle indicators in a CC high-risk cohort of WLWH and about upscaling screening and treatment services at the level of a rural referral hospital in Tanzania. Furthermore, it will provide exploratory data on novel assays.

**Trial registration:**

ClinicalTrials.gov Identifier: NCT05256862, date of registration 25/02/2022. Retrospectively registered.

**Supplementary Information:**

The online version contains supplementary material available at 10.1186/s12913-023-09113-3.

## Background

Cervical cancer (CC) most frequently arises from persistent infection with oncogenic high risk (HR-) human papillomavirus (HPV) types [1]. Due to global disparities in access to health care services and concurrent epidemiological risk factors (e.g., HIV infection), CC remains a major cause of morbidity and premature mortality in many low- and middle-income countries (LMICs). Despite availability of effective methods for prevention, early diagnosis and treatment [2–7], women living with HIV (WLWH) have a sixfold higher risk of acquiring HPV infection and developing cancerous lesions [8], even at younger ages [9, 10].

The World Health Organization (WHO) is leading a global effort to eliminate CC as a public health problem, proposing the “90–70-90” targets to meet by 2030: 90% of girls fully vaccinated against HPV by 15 years of age; 70% of women screened using a high-performance test by 35 years of age and again by 45 years of age; 90% of women identified with CC treated and given care [11].

The United Republic of Tanzania, where this study is planned, has the highest incidence of CC in East Africa, with 10,241 new cases diagnosed in 2020, a cumulative risk of 25.3% of new cases and 24.2% of cancer-related deaths per year according to the IARC/WHO Report [12]. Among WLWH, high-risk HPV (HR-HPV) positivity rates have been reported to be as high as 46.7% [13]. Cohorts based in Dar es Salaam, Moshi and Mwanza are currently also being investigated with regard to the distribution of different HPV genotypes [14].

CC is preventable with HPV vaccination. Following a successful pilot project in 2014 in the Kilimanjaro region among girls aged 9 – 14 years, in 2018 the Ministry of Health (MoH) of Tanzania introduced nationwide vaccination as a school-based program for 14-year-old girls [15], reaching an estimated coverage of 78% for the first dose and 49% for the second dose by the end of 2019 [16]. Nevertheless, the limitation of vaccination is the insufficient coverage of all HR-HPV types and a large and older non-vaccinated population, necessitating ongoing secondary prevention. In 2011, the MoH of Tanzania had successfully established a nationwide screen-and-treat program using speculum-aided visual inspection with acetic acid (VIA) and cryotherapy for precancerous lesions [15]. Given the higher risk for WLWH, screening is recommended at the time of HIV diagnosis and then annually [17], optimally integrated into routine HIV care, together with screening and treatment of sexually transmitted infections (STI) and female genital schistosomiasis (GS) [18].

VIA is used to identify acetowhite lesions, which if eligible according to the WHO guidelines, are treated with cryotherapy or thermal ablation.

While VIA, as well as cryotherapy for VIA positive (VIA +) minor changes can be provided effectively at a district level, many factors, including disease perception, stigma, equipment, and personnel availability can affect the uptake [15, 17]. Moreover, previous studies in Tanzania reported up to 30% of women with documented precancerous lesions not receiving cryotherapy immediately after diagnosis, and around 80% of them subsequently being lost to follow-up [15]. Further steps in the cascade of care, in the case of larger lesions or invasive cervical cancer (ICC), are hampered by additional factors including geographic confinement of cancer clinics as well as cost of service [15, 17]. Data on the number of women not receiving loop electrical excision procedure (LEEP) although indicated and/or not referred to cancer facilities once a diagnosis has been confirmed are unavailable.

In order to meet the WHO targets, higher coverage of screening services and scaling-up of existing screening points with more sensitive and specific methods such as HPV-testing, and therapeutic options for severe dysplasia/ICC such as LEEP within a same-day-screen & treat approach, is urgently needed [18–20]. As of 2021, HPV testing is already part of routine screening programs in 48 countries, mainly, but not exclusively, in Europe and America, thanks to the roll-out of molecular diagnostic platforms [21]. If performed on self-collected samples, it is a cost-effective and time saving approach which improves screening uptake due to better acceptance compared to physician collected samples [22–26]. Self-sampling has been proven effective in sub-Saharan African countries, with a reported sensitivity of 88–96.7% and specificity of 32.1–82.8% [27]. Moving to an HPV-based strategy has two major limitations: the same-day screen & treat approach depends on the analysis turnaround time of HPV tests, and secondly it has low specificity for severe dysplasia / ICC [28] in populations with high HPV prevalence. Based on this rationale, novel serological tests that target antibodies against HPV-oncogenic protein complexes and mRNA-based technologies targeting dysplasia-specific biomarkers are being explored in this study as well. These tests could allow direct identification of patients requiring treatment and spare the need for conventional pathological examinations, for which equipment and expertise is lacking in the country (Supplementary material: Annex A1 und A2).

## Methods/design

### Study design

This is a before-and after study to evaluate the impact and feasibility of upscaling CC screening and treatment services for WLWH attending a rural referral CTC in Tanzania. The main objective is to evaluate the uptake by WLWH attending screening after implementation of HPV testing on a self-sampled cervico-vaginal smear, compared to a retrospective cohort screened by VIA. HPV testing has been implemented in a bundle with: a) a smartphone integrating a mobile colposcope (EVA system, Mobile ODT, Israel) for digital enhancement of VIA examination with cervix magnification and second look/quality control; b) thermal ablation in place of cryotherapy (thus avoiding the need for replenishing nitrogen gas cartridges); and c) LEEP.

We adopted an uncontrolled before-and-after design to compare proportion of WLWH attending screening before and after implementing mentioned interventions. A sub-study with cross-sectional design aims to explore diagnostic performance of two novel tests: the first, QuantiGene-Molecular-Profiling-Histology (QG-MPH), is based on transcriptomic biomarker analysis, while the second is a serological assay to detect antibodies against HPV16-L1 [29], either with a qualitative (Prevo-Check®) or quantitative (PT Monitor®) approach (Table 1. Supplementary material Annex A1 and A2). Further objectives are to determine the adherence to recommendations after screening, to assess the prevalence of HPV genotype-specific infection as well as other co-infections, and to assess feasibility, acceptability and costs of the new implemented screening and treatment plan.

**Table 1:** Novel assays

Test	Name	Target	Description
Lateral Flow Assay (LFA)	PT Monitor® (Abviris GmbH, Germany)	HPV16-L1 Ab	Blood-based (serum) competitive immunoassay assessing the presence of epitope-specific antibodies against HPV16-L1. Elevated levels of these antibodies are associated with the presence of HPV16-induced cancer or pre-cancer. A quantitative readout is possible with an optical table-top reader (aLF reader by Qiagen, Germany). CE-marked IVD
Rapid Lateral Flow Assay (rLFA)	Prevo-Check® (Abviris GmbH, Germany)	HPV16-L1 Ab	Qualitative (yes/no) output of LFA (PT Monitor®) in form of rapid capillary point-of-care test with a cut-off of HPV16-L1 Ab > 1000 ng/ml. CE-IVD-marked for the detection of HPV16-induced anal and oropharyngeal cancers
Probe-based RNA Assay	HPV and dysplasia test – QuantiGene-Molecular-Profiling-Histology (QG-MPH)	mRNA of HPV oncogenes and cellular biomarkers	Cell-based. Quantitative detection of HPV16, 18, 26, 31, 33, 35, 39, 45, 51, 52, 53, 56, 58, 59, 66, 68, 73, 82 and cellular biomarkers, correlating with severity of a dysplastic lesion. The emergence and strength of biomarkers define the lesion stage. The QuantiGene 2.0 platform (ThermoFisher) is used 2 Experimental molecular IVD, Charité-University Hospital Berlin, DE (WO2020/161285 A1)

### Setting

The study is conducted in rural south-central Tanzania, at the St. Francis Referral Hospital (SFRH) in Ifakara, a 371-bed designated district referral hospital for the districts of Kilombero and Ulanga (Morogoro Region), serving over 800,000 inhabitants of the valley of Kilombero. Since its accreditation in 2005, a hospital-based HIV Care CTC was built up as a center for integrated care and as a research platform, the "Chronic Disease Clinic of Ifakara” (CDCI), through an international partnership between the hospital, the Ifakara Health Institute (IHI), the Swiss Tropical and Public Health Institute (Swiss TPH) and the University Hospital of Basel, Switzerland (USB) [28]. In addition to participation in the National AIDS Control Program (NACP) cohort, the CDCI has an open, prospective cohort, the Kilombero and Ulanga Antiretroviral Cohort (KIULARCO) [30] since 2008, where approximately 2500 women are currently under active care. At SFRH, CC screening by VIA is performed twice weekly at the Reproductive and Child Health clinic (RCH) and when indicated cryotherapy is offered in a single visit. Further investigations and treatments for VIA + women with major changes or suspicion of CC, including punch biopsy and hysterectomy, are payable services, while screening procedures are for free for WLWH, financed by the MoH and implementing partners of HIV programs and are also accessible at a modest cost (TSZ 500, USD 0,22) for HIV-negative women. For chemo- and/or radiotherapy, patients need to be referred to Ocean Road Cancer Institute (ORCI) in Dar es Salaam. Further opportunities are presented by outreach interventions in peripheral health centers, which are also supported by government implementation partners.

### Eligibility and enrollment

The study recruits consenting WLWH, aged 18 to 65 years, enrolled in KIULARCO, who have been on antiretroviral therapy (ART) for at least three months. Exclusion criteria consist of reported pregnancy, known CC and/or any other condition interfering with the visualization of the cervix, and previous hysterectomy. Participation in the study will be offered as per Standard Operating Procedure (SOP) during routine visits at the CDCI, where WLWH are referred either by the triage nurse or by the attending clinician, or directly at the CC screening service in case of self-referral. Specifically trained nurses will provide, in the native language of Kiswahili, detailed explanations and a copy of the informed consent form (ICF) to eligible WLWH, which will be read aloud to illiterate patients. Any questions or concerns will be answered accordingly. If the patient is willing to participate, the ICF will be signed or marked with their fingerprint and kept in the patient’s data file. Participants will undergo screening and treatment as specified in paragraph “Study procedures”. WLWH not eligible for the study but eligible for screening according to national guidelines, as well as any other women attending screening, will still benefit from digitally enhanced VIA and thermal ablation / LEEP if needed.

### Study procedures

#### Blood sampling

A rapid lateral flow assay (rLFA) (Abviris GmbH, Germany) to detect anti-HPV16-L1 antibodies will be performed first, at the point-of-care (POC), on a single drop of finger capillary blood (as described in Table 2), with qualitative readout. A positive result will address the participant to physical examination / colposcopy at the same visit. Then, a venous blood sample is drawn for a further quantitative read-out serological analysis (Supplementary Materials: Annex A1 und A2).

**Table 2:** Procedures

	Screening (day 0)	Baseline (day 0)	Follow-up (Day 1 up to 6 months)	Follow-up (6 months)
Informed consent procedure	X	X		
Eligibility check	X	X		
Case report form (REDCapTM)		X	X	X
Point-of-care blood test (capillary sample) ^a^		X		
Laboratory-based analysis^b^		X		
Cervico-vaginal smear self-sampling ^c^		X		X
Physical examination / colposcopy		X^d^	X	X^d^
Urine		X^d^		X^d^

^a^ PrevoCheck® with qualitative readout

^b^ Serum for PT Monitor® (quantitative analysis) and for Schistosomiasis antibodies

^c^ Seegene Anyplex ™ II 28 HPV, QG-MPH, STI and GS tests

^d^ Only if symptomatic, microscopy for detection of Schistosomiasis eggs

#### Self-sampling procedure

After instruction the participant performs cervico-vaginal self-sampling with the Evalyn® Brush (Rovers Medical Devices B.V., Oss, Netherlands) a medical device designed for this purpose and already validated in large scale screening programs [31, 32].

#### Physical examination / mobile colposcopy

**Fig. 1:**
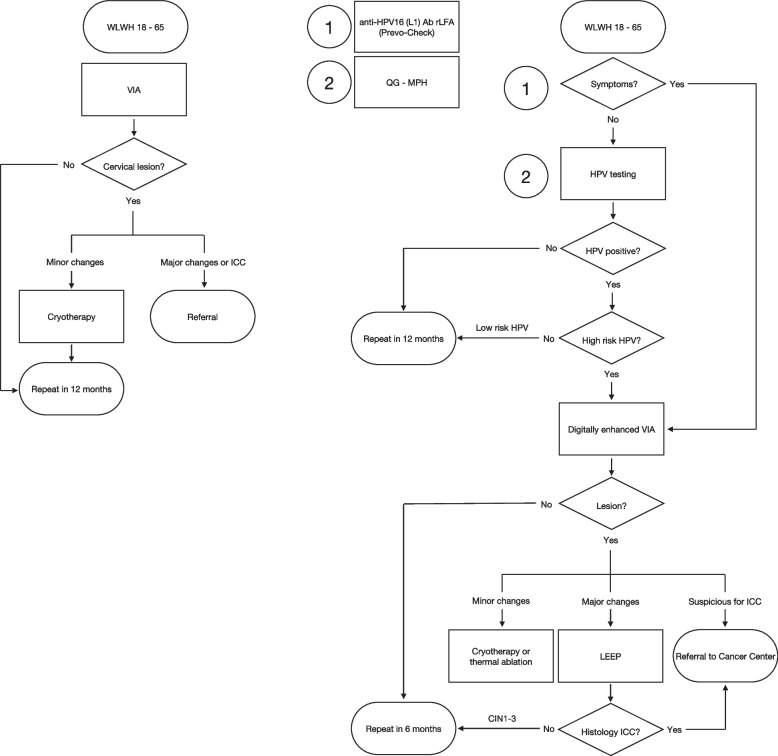
Study algorithm. Cervical cancer screening and treatment pathway at St. Francis Referral Hospital, Ifakara, TZ, before and after implementation of HPV testing. Circled numbers mark at which level could be applied novel tests used in the study. Legenda: Ab: antibody; CIN (1-3): Cervical Intraepithelial neoplasia; HPV: Human Papilloma Virus; ICC: invasive cervical cancer; LEEP: Loop Electrosurgical Excision Procedure; QG-MPH: Quantigene Molecular Profiling Histology; rLFA: rapid Lateral Flow Assay; VIA: Visualisation with acetic acid; WLWH: Woman living with HIV

The first participants will undergo physical examination and VIA at baseline visit, in accordance with the national guidelines, as well as HPV-testing as detailed further, until 150 HR-HPV negatives have been examined as a control population for the diagnostic sub-study. Thereafter, only participants that report vaginal symptoms (e.g., vaginal discomfort, abnormal vaginal discharge, pelvic pain, abnormal vaginal bleeding, suspected STI or CC) and/or test positive at rLFA will undergo physical examination at baseline. Otherwise, physical examination is reserved for participants with detected HR-HPV infection, and this will take place at a second visit, due to laboratory turnaround time. Physical examination is performed as per local routine, i.e., with speculum and VIA, in accordance to WHO/IARC standard recommendations [33]. Images are recorded with the aid of a mobile colposcope (EVA system®, MobileODT Ltd, Tel Aviv-Yafo, Israel). Lesion interpretation and classification will be done as specified in Annex A. If the lesion is compatible with an STI or GS, recommendations for treatment will be provided according to National Guidelines [17]. Precancerous lesions will be treated according to severity (Fig. 1 Study Algorithm; Supplementary materials: Annex B).

#### Laboratory analysis

The samples will be stored at room temperature and brought to the IHI laboratory, located next to the SFRH, at the end of the day. Serum will be obtained from the whole blood sample for quantification of antibodies to the HPV16-L1 DRH1 epitope (PT Monitor®, Abviris, Germany). After pre-incubation of 25 µL serum with an HPV16-L1-specific reagent for 5 min, the mixture is transferred onto a lateral flow test cassette. The test result is measured 15 min later with a table-top reader (Qiagen, Germany).

Self-collected cervico-vaginal smear samples are used for HPV testing and QG-MPH, an innovative patented assay by Charité-Universitätsmedizin Berlin (Charité), Berlin, Germany (Charité Technology Transfer, patent WO2020/161285 A1) based on QuantiGene 2.0 (ThermoFisher Inc., Waltham, MA, USA) platform technology. QG-MPH has promising results for detecting CIN3 + with 90% accuracy (i.e., high sensitivity and specificity), and could become a quick onsite-triage method and a pathology-replacing, molecular diagnostic assay [34]. Further details are provided in Annex A2.

The cervico-vaginal swab is fixed in a methanol-based transport medium (ThinPrep® PreservCyt® solution, Hologic N.V., Belgium). HPV testing takes place in two steps: DNA extraction will be performed at IHI Laboratory in Ifakara, where samples undergo temporary storage at -20 °C. Every 1–2 weeks, a batch of 200–400 samples will be transported to the IHI Laboratory in Bagamoyo for PCR-based DNA amplification with the Seegene Anyplex™ II 28 HPV Detection Test (Seegene Inc., Korea), an RT-qPCR assay designed to detect and individually genotype 19 different HR-HPV types (HPV 16, 18, 26, 31, 33, 35, 39, 45, 51, 52, 53, 56, 58, 59, 66, 68, 69, 73 and 82) and 9 low risk (LR)-HPV genotypes (i.e. HPV 6, 11, 40, 42, 43, 44, 54, 61 and 70) in 2 parallel wells. Staff training and laboratory setup for transcriptome analysis in Tanzania was recently hindered by the SARS-CoV-2 pandemic. CFX96™ Dx Real-time PCR System was not lent to the IHI branch of Ifakara because of shortages of the instrument in East Africa. A cervico-vaginal smear sample aliquot will also be shipped for further transcriptome analysis at the HPV laboratory of Charité according to the MTA (Material Transfer Agreement).

Tissue of cervical biopsies and/or LEEP excisions will undergo histopathological studies at ORCI. Urine sediment microscopy for *Schistosoma* egg detection for GS will be performed at the IHI laboratory in Ifakara.

#### Therapy

VIA + women with minor changes are treated at the same session by thermal ablation**,** using a C3 Thermo-coagulator (WISAP Medical Technology, Germany), an easy to handle, reusable, thermo-probe device, which can run on the standard power grid or be connected to an external powerpack, and thus practical in settings where the electricity supply might be interrupted. Similar devices are available at other facilities in the region, the nearest being Morogoro Regional Hospital (ca. 231 km or five hours drive from the study site).

VIA + women with major changes and/or suspicion of ICC and/or transformation zone type 3 are treated at the same visit in a separate session in the gynecology surgical theater by LEEP. LEEP is a 10-min procedure to remove a cone-shaped area of tissue from the cervix for diagnostic and/or therapeutic purposes with a thin wire loop heated by electricity. It has the potential of being curative, usually without significant adverse events [35]. Excised tissue will be sent for histopathological analysis. LEEP surgery is already available at several facilities in Tanzania, the nearest being in Dar es Salaam (ca. 400 km or eight-ten-hours drive from the study site).

#### Follow-up

The follow-up plan is determined by the analysis results (Fig. 1). If antibodies to HPV16-L1 and/or HR-HPV DNA are detected in baseline samples, the participant will be contacted within 7–14 days by phone or, if unreachable, personally notified by a community health worker, in order to plan the physical examination at the patient’s earliest convenience (either at the next CTC appointment or at an extra visit). Independently from these results and any further treatment (LEEP or thermal ablation), participants who have antibodies to HPV16-L1 and/or HR-HPV detected at baseline will be asked to repeat cervico-vaginal swab collection for Anyplex™ II HPV28 Detection and QG-MPH after 6 months. If baseline samples are negative for HR-HPV and for antibodies to HPV16-L1, re-screening will be recommended after 12 months according to the NACP guidelines.

### Endpoints

The primary endpoint is screening coverage, defined as the proportion of WLWH enrolled in KIULARCO screened for CC in the 12 months period after implementation of HPV testing. Participants that undergo screening and transfer out to other CTC within the study period are also included. Secondary endpoints are listed in Table 3.

**Table 3:** Endpoints

	Endpoint
**HPV prevalence & persistence**	Proportion of participants with documented HR-HPV, LR-HPV and HPV negative at baseline
	HPV genotypes at baseline
	Proportion of participants with documented HR-HPV, LR-HPV and HPV negative at follow up (6 months after the first sample)
	HPV genotypes at follow up (6 months after the first sample)
	Rate of clearance and persistence of HPV at 6 months follow-up and factors associated with viral persistence
**Cervical lesions**	Prevalence of cervical dysplasia among the patients with HR-HPV
	Prevalence of co-infection (STI and GS)
**QG-MPH® performance**	Prevalence of HSIL/CC detected by QG-MPH® in cervical smear samples
	Diagnostic test accuracy (sensitivity, specificity, PPV, NPV) in detecting patients with CIN3 + compared to colposcopy images, HPV infection detected by Seegene Anyplex ™ II 28 and histology
**Prevo-check® performance**	Prevalence of HPV16 positive CIN2 + detected by Prevo-check®
	Diagnostic test accuracy (sensitivity, specificity, PPV, NPV) in detecting patients with HPV 16 compared to Seegene Anyplex ™ II 28
**PT Monitor® performance**	Comparison of HPV16-antibody levels in women with and without HPV16-positive CIN2 + . Calculation of test performance parameters (sensitivity, specificity, PPV, NPV) at different antibody thresholds and calculation of optimum cut-off for CIN2 + detection by ROC analysis and Youden index
	Diagnostic test accuracies (sensitivity, specificity, PPV, NPV) in detecting patients with HPV16-induced CIN2 + by PT Monitor® compared to colposcopy images, HPV infection detected by Seegene Anyplex ™ II HPV 28 Detection and histology
**Screening strategy implementation**	Acceptability of cervical self-sampling
	Acceptability of proposed implemented pathway
	Adherence to follow-up
	Rate of WLWH completing the CC screening and treatment care plan
	Prevalence of cryo/thermoablation-non-eligible lesions treated with LEEP
	Service accessibility (means of travel to the point of screening: foot, car bajaj, public transport bus; distance to hospital < 5 km, 5—10 km, > 10 km, perceived difficulty of travel yes/no; mean of waiting time in minutes at point of screening)
	Social and educational status of WLWH attending and refusing CC screening as well as their awareness regarding HPV infection, transmission, risk behavior, CC
**Costs**	Direct and indirect costs associated with CC screening and treatment before and after the proposed implementation was adopted including the bundle of interventions
	Direct and indirect costs, as well visit durations, for the current VIA and cryotherapy standard of care and for the implemented path with HPV-based triage

*Abbreviations: CC* Cervical cancer, *CIN* Cervical intraepithelial neoplasia, *GS* Female genital schistosomiasis, *HSIL* High Grade Squamous Intraepithelial Lesion, *HPV* Human Papilloma Virus, *HR* High-risk, *LFA* Lateral Flow Assay, *LR* Low risk, *NPV* Negative predictive value, *RNA* Ribonucleic acid, *QGMPH* QuantiGene-Molecular-Profiling-Histology, *STI* Sexual transmitted infection, *PPV* Positive predictive value, VIA Visual Inspection with Acetic Acid, *WLWH* Women living with HIV

### Data collection and management

Attendance to screening and its results are routinely recorded on a card given to the patient, and on paper registries. For the purpose of this study, data are additionally recorded in an online, password-protected electronic data capture software (REDCap™). The questionnaires administered to the participants will be filled out in real time by study nurses or physicians on a tablet device, while laboratory data will be entered by the laboratory technicians as soon as they become available. Data relevant for patient care will be recorded separately in the electronic database of KIULARCO (OpenMRS v2.0.6). Participant data will be managed confidentially: pseudonymized (study ID as well as NACP ID for linkage with NACP and KIULARCO dataset) for the purpose of patient care and anonymized for scientific evaluation and further dissemination of results. At baseline, sociodemographic data, medical history, laboratory analysis results as well as knowledge, attitude and practice as regards CC and HPV vaccine will be captured. A data safety and management board is not planned. Data monitoring will be performed on a regular basis by co-investigators not involved in data collection. At the end of the project, the entire database, property of IHI and USB, will be archived for minimum period of 10 years. The study represents implementation research: HPV testing and established therapeutic interventions (thermal ablation, LEEP) are already available for routine care elsewhere in the country. The experimental diagnostic tests have a minimal impact (only capillary POC test) on clinical decisions and have been granted approval by TMDA. Adverse events will not be recorded. A Data Transfer Agreement (DTA) has been signed between IHI and respectively Charité and USB.

### Statistical analysis

For the primary endpoint, the hypothesis is that implementing a bundle of interventions comprising HPV testing on self-collected samples will improve rates of CC screening among WLWH enrolled in KIULARCO from 8 to 30%. Due to the uncertainty about the WLWH population size, this hypothesis is considered as exploratory, therefore only the precision of the proportion is estimated (screening rate), with a 95% confidence interval. The proportion of WLWH attending CC screening in the post-period will be estimated with 95% confidence interval.

For the secondary and other endpoints no hypothesis will be formulated and results will be compared in an exploratory manner. Diagnostic test accuracies (sensitivity, specificity, positive predictive value and negative predictive value) will be calculated for comparison. Accuracies will be calculated with a 95% confidence interval. Prevalence of secondary endpoints will also be estimated with 95% CI's. Diagnostic tests will be pairwise compared using McNemar test. The assessment of acceptability and feasibility will be evaluated with exploratory statistics. The agreement of raters between diagnostic interpretations will be described with kappa coefficients and polychoric correlations. All secondary parameters will be reported with descriptive statistics including minimum and maximum values, interquartile range, median, mean, SD, count, and proportion as appropriate. Comparisons between study groups will be done using Kruskall-Wallis test for metric or ordinal data and Fisher's exact test for categorical data. A *p*-value < 0.05 (two-sided) is considered as statistically significant.

All evaluations will be done with the latest version of the statistical software R.

### Sample size

No sample size calculation was done due to the exploratory nature. For the primary outcome, we performed an estimate of the precision. Given a precision of ± 3% and assumed proportion of 30% self-sampling (enrolled subjects), a sample size of 1200 WLWH estimates a 95% confidence interval completely within the required precision. In the pessimistic case with a given a precision of ± 2% and assumed proportion of only 10% self-sampling (enrolled subjects), a sample size of 1200 WLWH estimates a 95% confidence interval completely within the required precision.

### Biological material

Serum and cervico-vaginal smear samples will be labelled, processed, analyzed, stored at the IHI laboratory in accordance with SOPs. For the QG-MPH test and quality control of HPV testing, one cervico-vaginal smear sample per participant will be transported from Ifakara to the HPV Laboratory, Charité (DE) as per the MTA. Once all analyses have been performed, the materials will be stored for a period of 10 years and thereafter destroyed by standard internal procedures of the IHI and Charité, respectively.

### Patient and public advisor

No patient or patient advisor was involved with study design, recruitment, or conduct.

### Study status in December 2022

Training in colposcopy and the routine use of a mobile colposcope started in February 2020, and HPV testing on self-cervico-vaginal collected samples was implemented starting from 30.07.2021. LEEP was introduced in September 2020 and thermal ablation in October 2021. As per 31.12.2022, 1500 participants have been enrolled.

## Discussion

This implementation project represents a micro-initiative, at rural referral hospital level, to contribute to the efforts of improving CC prevention and the care cascade in Tanzania, complying with the WHO and national recommendations regarding secondary and, in part, tertiary prevention. The choice of implementing treatment procedures along with HPV testing has an ethical rationale. The decision to substitute the already in-use cryotherapy with a thermal ablation device was made after frequent reports of cryotherapy gas supply challenges, with consequently missed treatment opportunities. The introduction of a mobile digital colposcope had the purpose of allowing second-look evaluations by collaborating clinicians, for continuous education and for quality improvement. The choice of a laboratory-based HPV testing platform over the on-site available Xpert® platform was firstly due to concurrent testing needs (esp. for tuberculosis diagnostics) which would have hampered an HPV-based single-visit screen-and-treat approach only, and secondarily because the chosen platform allows for identifying more individual genotypes. The choice of a before-and-after study design has certain limitations, in that the intervention effect may be overestimated by unrecorded, concurrent factors (e.g., in this case: increased patient sensitization to CC, training of personnel, screening offered 5 days a week instead of twice weekly, and financial incentives to staff performing screening), which in this case are relevant to some extent, since the ideal comparison is the WHO’s second target (70% screened). Further, the low estimates of pre-intervention screening rates (8%) may reflect incomplete reporting or heterogeneous data sources. For the purpose of this study, a digital case report form (e-CRF) was developed which could be accessed via a tablet device or computer (also provided) and filled out in real-time. This should minimize bias in data collection. Considering that CC screening was already routinely offered to the study population and that reporting may be of suboptimal quality, recall bias cannot be ruled out for the patients who had previous diagnosis of precancerous lesions, although this should not affect the primary outcome. Cost of service is a major concern regarding the sustainability of the implementation project, which we plan to address in a future publication. Provided free of charge or after a symbolic financial contribution, CC screening and treatment with VIA / cryotherapy was financially supported by non-governmental implementing partners, including financial incentives to the screening team and an additional outreach program. Currently, diagnostic and therapeutic options are limited in peripheral health-care centers of Tanzania and different factors hinder access to CC screening and treatment programs. Implementing “near-home” options may substantially increase the participation rate as well as improve the adherence to the appropriate care plan, with rationalization of health-care center resources and improvement of quality of services.

In addition, the study has the potential to understand the specific needs of a semi-rural patient population (including sexual, reproductive and HIV-related aspects), of outcome of screening and treatment, and reasons for non-adherence, which are important factors for future scaling-up procedures. The results of this study will provide additional information to the Ministry of Health of Tanzania on the epidemiology of HPV infection, CC and comorbidities in the high-risk population of WLWH accessing a rural referral hospital. It might help to provide elements useful for the development of strategies in similar settings for comprehensive reproductive and sexual health care. Since the study team was already involved in CC prevention and treatment, and the provided instruments will be available for routine clinical care, patients not included in this study are expected to benefit as well. Finally, the exploratory analysis on novel tests will hopefully contribute to potential innovations in clinical practice, which, if proven effective, may contribute to a reduction in CC screening time and costs worldwide.

## Supplementary Information

**Additional file 1: Supplementary Materials - Annex A.** Annex A. Details and evidence on novel diagnostics. Annex A1. HPV16-L1 immunoassay (PrevoCheck® and PT Monitor®). Annex A2. QuantiGene Molecular Profiling Histology (QG-MPH®).

**Additional file 2: Supplementary Materials - Annex B.** Annex B. Definitions.

## Data Availability

Not applicable.

## Abbreviations

CC: Cervical cancer
CDCI: Chronic Disease Clinic of Ifakara
Charité: Charité University Hospital in Berlin
e-CRF: Case report form
CTC: Care and treatment center
DTA: Data Transfer Agreement
HPV: Human papillomavirus
HR-HPV: High-risk HPV
LR-HPV: Low-risk HPV
ICF: Informed consent form
IHI: Ifakara Health Institute
KIULARCO: Kilombero and Ulanga Antiretroviral Cohort
LEEP: Loop Electrosurgical Excision Procedure
MTA: Material transfer agreement
rLFA: Rapid Lateral Flow Assay
NACP: Tanzania’s National AIDS Control Program
POC: Point-of-care
QG-MPH: QuantiGene-Molecular-Profiling-Histology Assay
RCH: Reproductive and Child Health clinic
SFRH: St. Francis Referral Hospital (setting)
VIA: Visual inspection with acetic acid
VIA +: Via positive
WLWH: Women living with HIV
