# Supplementary material for: Upscaling cervical cancer screening and treatment for women living with HIV at a rural referral hospital in Tanzania: protocol of a before-and-after study exploring HPV testing and novel diagnostics
Source: BMC Health Serv Res. 2023 Mar 10;23:234. doi: 10.1186/s12913-023-09113-3 (PMC9998252; doi:10.1186/s12913-023-09113-3)
Supplement: Supplementary file 1 — Additional file 1: Supplementary Materials - Annex A. Annex A. Details and evidence on novel diagnostics. Annex A1. HPV16-L1 immunoassay (PrevoCheck® and PT Monitor®). Annex A2. QuantiGene Molecular Profiling Histology (QG-MPH®). [file 12913_2023_9113_MOESM1_ESM.docx]

**Annex A**

1. **Details and evidence on novel diagnostics**
   1. **HPV16-L1 immunoassay (PrevoCheck**® **and PT Monitor**®)
   2. **QuantiGene-Molecular-Profiling-Histology (QG-MPH**®**)**
2. **Details and evidence on novel diagnostics**

**1. HPV16-L1 DRH1 epitope-specific immunoassay (Prevo-Check® and PT Monitor®, Abviris GmbH, Germany)**

HPV infection constitutes a risk but not proof for HPV-associated disease, thus raising questions on the discriminatory capacity of classical serological assays regarding pre-cancerous and cancerous lesions^1^. Nevertheless, antibodies directed against specific viral proteins epitopes of HPV16, the most carcinogenic of HPV subtypes, are under study with various results^1^. Abviris developed a competitive immunoassay to assess the presence of epitope-specific antibodies against HPV16-L^1^. This antigen stimulates production of antibodies to HPV16-L1 when dysplasia progresses to high-grade pre-cancer or CC^1^. It can be used in patients who have not been vaccinated against HPV, and the presence of higher antibodies titers correlates with the presence of pre-cancerous lesions or cancer. The assay is available in the form of a rapid lateral flow test (Prevo-Check®) which indicates whether anti-HPV16 antibodies are present in capillary blood above a pre-set threshold, with a yes/no readout provided in 20 minutes. In addition, quantification of antibodies can be achieved by using a lateral flow test (PT Monitor® on serum, coupled to a table-top reader (aLF reader by Qiagen, Germany) which converts line intensities into antibody concentrations based on an internal calibration function (Fig. 1). Prevo-Check® in the European Union is marketed for the early detection of HPV16-induced head and neck squamous cell carcinoma (HNSCC) as well as anal cancers. Performed in 1500 subjects, it had a specificity of 99.3% (95% CI 98.6% - 99.8%) in the cancer-relevant age group of the apparently healthy population (≥30 years, n=895), and a sensitivity at the time of diagnosis of 95% in patients with oropharyngeal cancer (n=20). For HPV16-induced anal carcinomas in people living with HIV (PLWH), sensitivity was 90% in the year before tumor diagnosis. The accuracy of Prevo-Check® results compared to clinical status was 99.1% (95% CI 98.3%-99.6%). In addition, similar accuracy was calculated performing PT Monitor® at an antibody threshold (cut-off) of 1000 ng/mL^1^. In subsequent studies, Prevo-Check® had 100% accuracy (sensitivity and specificity) for HPV16-induced oral cavity carcinomas^2^. It has also been assessed in PLWH to detect anal dysplasia with a statistically significant association between the level of the measured antibody titer and both the likelihood and severity of an abnormal cytology finding^3^. For antibody titers above 1000 ng/ml (equivalent to Prevo-Check® cut-off), the positive predictive value (PPV) for an abnormal anal cytology finding was 0.88; specificity was 97%. By contrast, the HR-HPV DNA test had lower PPV and specificity (0.64 and 61%, respectively). At the 1000 ng/ml antibody cut-off, the serological assay detected 56%, 25%, and 7% of all anal HSIL, ASCUS, and LSIL cases, respectively. Lowering the cut-off to 200 ng/ml allowed the identification of 100% HSIL, 50% ASCUS, and 64% LSIL cases. The ROC curve for anal HSIL detection was 0.92 (95% CI, 0.84–1.00), without significant effects of recent CD4+ cell count on HPV16 L1 serology^3^. It may also be used for the detection of precancerous cervical lesions as well as for post-treatment biomarker, post-therapy monitoring and early diagnosis of relapse.

1. **QuantiGene-Molecular-Profiling-Histology (QG-MPH)**

QG-MPH combines mRNA-based HPV detection by E6 and E7 oncogene expression of 18 HR-HPV genotypes (HPV16, 18, 26, 31, 33, 35, 39, 45, 51, 52, 53, 56, 58, 59, 66, 68, 73, 82) with expression of sets of biomarkers newly expressed or up-regulated during progression (dysplasia detection and staging), reducing the risk of low specificity in high HPV prevalence areas, younger age groups and risk groups like WLWH with a high HPV prevalence of infection. The WHO recommends screening for 12 genotypes with higher carcinogenic potential. WLWH, however, have faster progression and also the less carcinogenic genotypes that rarely cause cancer in healthy women more frequently induce transformation in WLWH. Therefore, these probable and potential carcinogenic genotypes (HPV26, 53, 66, 68, 73, 82) were included in this innovative assay. Secondly, commercially available PCR-based assays cannot quantify the viral load or activity of an HPV infection and thus cannot discriminate between transient, latent, active, transforming or progressed infections or underlying dysplasia. QG-MPH, by detecting mRNA of the viral oncogenes and quantitating the expression strength, identifies active infections and dysplasia severity correlates with higher expression strength. The addition of stage-specific biomarkers helps and allows addition of the rarely carcinogenic genotypes as the specificity of the assay for CIN2+ detection is not reduced by the less carcinogenic genotypes.

This test might offer the possibility for primary CC screening and substitution of VIA by discriminating between cases of HPV positivity, mild and severe dysplastic cervical lesions and CC in settings lacking pathology laboratory equipment and expertise. It is performed on a self-taken cervical smear sample with the Evalyn® Brush (Rovers Medical Devices, Oss, The Netherlands) or on a physician-taken cervical smear and is more accurate in determining CIN3+ with a sensitivity of 0.83 (0.36; 1.00) and specificity of 0.82 (0.69; 0.91)^4-5^ compared to PCR-based HPV tests that have a very high sensitivity (>95% for CIN2+ detection) but a low specificity (<20% due to transient HPV infections). Diagnosis with QG-MPH has been calculated to cost approximately less than €100 (internal calculation and pers. communication) as compared to standard of care with costs up to €565 in a German setting, depending on the diagnosis. The cost of the assay is in the range of PCR-based HPV tests. Time to definitive result is shortened from 2-6 weeks (according to the center capacity) to 2 days with only one visit needed and obtained from the first screening smear. QG-MPH is based on QuantiGene 2.0 (ThermoFisher Inc. Waltham, MA, USA) an alternative mRNA detection and quantification platform technology to qRT-PCR. Initial studies suggest that the QuantiGene 2.0 platform allows an efficient expression analysis within 36 hours, performed on a 96-well plate, making it high-throughput capable. A standard cervical smear is lysed for mRNA liberation, then processed as an ELISA-like assay, and a robust reader (Magpix, Luminex Inc., Austin, TX, USA) delivers the results, which consist of HPV genotype (risk of progression), cellularity of the sample (quality control and normalization), biomarker strength (up-regulated with transformation and progression), and biomarker neo-expression (invasive stage). Using exclusively sequenced hybridization and signal amplification, the assay avoids enzymatic reactions and target amplification that often bears the risk of cross-contamination. The assay format has been patented by Charité-Universitaetsmedizin Berlin, Germany (Charité Technology Transfer, patent WO2020/161285 A1) and is under further validation in studies. Due to the patenting processes, the publication of sensitive data was not possible until now. Initial studies have shown the ability of this test to differentiate between 18 individual HR-HPV genotypes and the dysplasia stages CIN2+, CIN3+, and CC. Implementation of the assay in a population-based screening study in Butajira, Ethiopa, detected in a pilot study three patients with dysplastic disease that were identified after HPV-based screening, VIA and colposcopy-based triage, and biopsy/surgery. Importantly, in comparison to PCR-based HR-HPV testing, the screening effort was reduced by 300-fold and the triage effort by 50-fold (manuscript in preparation, not yet published). The accuracy of the risk scores as determined by QG-MPH were compared to the standard of care (HPV DNA PCR Test; PAP and liquid-based cytology). QG-MPH had the best accuracy concerning sensitivity (83%) and specificity (82%), while liquid-based cytology (LBC)/PAP smear had a sensitivity of 33% and specificity of 80%, and HPV PCR a sensitivity of 83% and a specificity of 58% to detect CIN3+ defined by histology ^4-5^. In the frame of this study, the use of QG-MPH aims to complement HPV testing, VIA triage and finally histology data, especially for WLWH who had received thermal ablation and from whom no tissue for definitive pathology diagnosis could be obtained.

**References**

1. Weiland, T. et al. DRH1 – a novel blood-based HPV tumour marker. EBioMedicine 56, (2020).

2. Blatt, S. et al. Clinical efficacy of an antibody-based detection system for human papilloma virus infection in oral squamous cell carcinoma. Clinical Oral Investigations 25, (2021).

3. Ecke, S., Huber, A., Hilfrich, R., French, L. &amp; Rheinholz, M. HPV16 L1 serological test shows high concordance with anal cytology in people living with HIV. Journal of Investigative Dermatology Innovations in press, (2022).

4. QG 2.0 assay handbook [accessible at: https://www.thermofisher.com/de/de/home/life- science/gene-expression-

analysis-genotyping/quantigene-rna-assays/quantigene-plex- assay.html (last access April, 24th 2022)]

5. QG-MPH patented under number: WO2020/161285 A1 “A method for determining the severity or grade of human papilloma (HPV) induced dysplasia’’
