# Supplementary material for: Upscaling cervical cancer screening and treatment for women living with HIV at a rural referral hospital in Tanzania: protocol of a before-and-after study exploring HPV testing and novel diagnostics
Source: BMC Health Serv Res. 2023 Mar 10;23:234. doi: 10.1186/s12913-023-09113-3 (PMC9998252; doi:10.1186/s12913-023-09113-3)
Supplement: Supplementary file 2 — Additional file 2: Supplementary Materials - Annex B. Annex B. Definitions. [file 12913_2023_9113_MOESM2_ESM.docx]

**Annex B. Definitions**

| **Definition** | **Features** |
| --- | --- |
| **VIA-positive* (VIA+)**1 | - Distinct, well defined, dense acetowhite areas with regular or irregular margins, close to the squamocolumnar junction (SCJ) in the transformation zone (TZ) or to the external os, if the SCJ is not visible; - Strikingly dense acetowhite areas in the columnar epithelium; - Entire cervix turning densely white; - Leukoplakia close to the SCJ; - Condyloma turning intensely white). |
| **VIA+ eligible for cryotherapy/thermal ablation**2 | - Mild dysplasia, that could be covered by the probe used; - TZ 1 and 2 TZ fully visible; - Whole lesion visible or not extending into the endocervix; - No suspicion of invasive or glandular disease (i.e., adenocarcinoma or adenocarcinoma in situ); - Probe tip available will achieve complete ablation of the SCJ epithelium, i.e., where it can reach the upper limit of the TZ (sometimes the SCJ can be seen high in the canal but a probe tip would not reach it). |
| **VIA+ eligible for LEEP** | - Moderate and severe dysplasia; - Glandular disease (i.e., adenocarcinoma or adenocarcinoma in situ); - Endocervical TZ, not fully visible (TZ 3). |
| **Transition zone (TZ)**1,3 | - TZ 1 is completely ectocervical and fully visible. - TZ 2 is partially endocervical, but is still fully visible. It may be shallow and within range of an ablative probe or may extend beyond reach of an ablative probe. - TZ 3 extends out of view up the endocervical canal, i.e., the SCJ, and is not fully visible. |

*** To be intended after application of acetic acid. SCJ = squamo cellular junction; TZ = transition zone**

**References**

1. A practical manual on visual screening for cervical neoplasia. IARC Technical Publication No 41. (2003). [accessible at Https://screening.iarc.fr/viavilichap2.php (last access Mai, 7th 2022)]

2. WHO guidelines for the use of thermal ablation for cervical pre-cancer lesions. Geneva: World Health Organization; 2019. License: CC BY-NC-SA 3.0 IGO. accessible at https://apps.who.int/iris/bitstream/handle/10665/329299/9789241550598-eng.pdf (last access Mai, 7th 2022)

3. Colposcopy and treatment of cervical intraepithelial neoplasia: a beginners’ manual. (2003). Accessible at https://screening.iarc.fr/colpochap.php?chap=2 (last access Mai, 7h 2022)
